# Potential therapeutic biomolecules of hymenopteran venom against SARS-CoV-2 from Egyptian patients

**DOI:** 10.1038/s41598-024-65038-9

**Published:** 2024-07-04

**Authors:** Eman A. Abd El Maksoud, Magda H. Rady, Ahmed Gad Taha Mahmoud, Dalia Hamza, Mohamed G. Seadawy, Eman. E. Essa

**Affiliations:** 1 Armed Forces Laboratories of Medical Research, El-Khalifa El-Maamoun, Mansheya El-Bakry, Heliopolis, Cairo Governorate Egypt; 2 Entomology Department Faculty of Science, Ain Shams University, Cairo, 11566 Egypt; 3 Microbiology Department, Armed Forces Laboratories of Medical Research, El-Khalifa El-Maamoun, Mansheya El-Bakry, Heliopolis, Cairo Governorate Egypt; 4 Zoonoses Department, Faculty of Veterinary Medicine, Cairo University, Giza, Egypt; 5 Biological Prevention Department, Chemical Warfare, Egypt Army, Cairo, Egypt; 6 Entomology Department, Faculty of Science, Ain Shams University, Cairo, Egypt

**Keywords:** Computational biology and bioinformatics, Drug discovery, Zoology, Entomology, Antimicrobials, Microbiology, Virology, SARS-CoV-2, Infectious diseases, Viral infection

## Abstract

The therapeutic potential of insect-derived bioactive molecules as anti-SARS-CoV-2 agents has shown promising results. Hymenopteran venoms, notably from *Apis mellifera* (honeybee) and *Vespa orientalis* (oriental wasp), were examined for the first time in an in vitro setting for their potential anti-COVID-19 activity. This assessment utilized an immunodiagnostic system to detect the SARS-CoV-2 nucleocapsid antigen titer reduction. Further analyses, including cytotoxicity assays, plaque reduction assays, and in silico docking-based screening, were performed to evaluate the efficacy of the most potent venom. Results indicated that bee and wasp venoms contain bioactive molecules with potential therapeutic effects against SARS-CoV-2.Nevertheless, the wasp venom exhibited superior efficacy compared to bee venom, achieving a 90% maximal (EC_90_) concentration effect of antigen depletion at 0.184 mg/mL, in contrast to 2.23 mg/mL for bee venom. The cytotoxicity of the wasp venom was assessed on Vero E6 cells 48 h post-treatment using the MTT assay. The CC _50_ of the cell growth was 0.16617 mg/mL for Vero E6 cells. The plaque reduction assay of wasp venom revealed 50% inhibition (IC_50_) at a 0.208 mg/mL concentration. The viral count at 50% inhibition was 2.5 × 10^4^ PFU/mL compared to the initial viral count of 5 × 10^4^ PFU/mL. In silico data for the wasp venom revealed a strong attraction to binding sites on the ACE2 protein, indicating ideal interactions. This substantiates the potential of wasp venom as a promising viral inhibitor against SARS-CoV-2, suggesting its consideration as a prospective natural preventive and curative antiviral drug. In conclusion, hymenopteran venoms, particularly wasp venom, hold promise as a source of potential therapeutic biomolecules against SARS-CoV-2. More research and clinical trials are needed to evaluate these results and investigate their potential for translation into innovative antiviral therapies.

## Introduction

Human coronaviruses are a major threatening issue for worldwide public health. SARS-COV-2 is the causative agent of the potentially fatal disease COVID-19^1^. Since the start of the pandemic in 2019, public health and social measures (PHSM) have been put into place all over the world to stop the spread of severe acute respiratory syndrome coronavirus SARS-CoV-2 and its related mortalities (World Health Organization, 2020). The update of the World Health Organisation (WHO) on 2 August 2023 was 768 983 095 confirmed cases and 6 953 743 confirmed deaths to SARS-CoV-2 infection. SARS-CoV-2 is a member of the coronavirus family. The membrane, spike, nucleocapsid proteins, and envelope are the four visible structural proteins found in SARS-CoV-2. The structural proteins that surround the RNA of the SARS-CoV-2 virus are responsible for its structural integrity^2^.

Serological tests and testing based on nucleic acids are the two primary categories for SARS-COV-2 detection^3^. The nucleocapsid and spike protein from SARS-CoV-2 have recently been used in serological diagnostic tests via ELISA, immunofluorescence, and a lateral flow test. Patients become seropositive 10 to 18 days following the beginning of symptoms, according to one study that used ELISA to test just antibodies to the nucleocapsid protein^2^.

In COVID-19 patients necessitating intensive care, Severe COVID-19 cases may involve "cytokine storms," where excessive inflammatory molecules damage lungs and other organs. The exact mechanisms driving these storms remain under active research^4^. In COVID-19, the SARS-CoV-2 spike protein binding to the ACE2 receptor triggers a chain of events leading to life-threatening hyperinflammation. This heightened immune response, characterized by an overproduction of inflammatory molecules, is similar to cytokine release syndrome (CRS) and can cause severe complications^4^.

Vaccination efforts are currently in progress across numerous countries; however, achieving 100% effectiveness is unlikely. While certain medications have been identified to reduce the severity of the disease, they do not guarantee improved outcomes in all cases^2,4^ .Therefore, the development of new medications is urgently needed. Plants' ability to provide medicinally relevant compounds for drug development has received a lot of attention. However, very few, if any, institutions labs that primarily looking at insects as potential drug sources. Moreover, there has been limited research on the potential of insect chemical biodiversity to contribute to biomedical sciences, a field referred to as pharmaceutical entomology. This topic has only been addressed in a handful of publications. Insects are the "new frontier for drug discovery and other natural product chemistry and can serve as a valuable source of prototype drugs^5,6^. Recent progress concerned with three powerful trends analytical chemistry, improved disease models, and rising threats from unknown pathogens—make it crucial to explore insects (arthropods) more deeply as potential sources of new drug^7^.

Among the insects' potentially helpful compounds are the toxins used to defend against attack by predators and other hazards, naming insect venom, which holds great promise for the future of natural products and medication development^8^. The second largest order of insects is order Hymenoptera, which comprises two venomous insects, bees and wasps. Hymenoptera encompasses the Apidae family, housing numerous bee species, including the prevalent honey bee (*Apis mellifera* L.), and the Vespidae family, which includes a diverse array of wasps. Notably, the oriental hornet (*Vespa orientalis*; Linnaeus, 1771) is localized in North Africa^9^. Hymenopteran venoms incorporate physiologically active proteins and peptides, including phospholipases, hyaluronidase, phosphatase, glucosidase, serotonin, histamine, dopamine, noradrenaline, and adrenaline, which are mixed in complicated ways in the venoms of bees and wasps^10^.

However, specific peptides unique to each insect species, such as apamin, melittin, and mast cell degranulating (MCD) peptides, are unique to bees. Wasps have their exclusive peptides, including mastoparan and bradykinin. These constituents act as ion channel agonists, antagonists, or neurotransmitters^11^. Recent literature showed promising evidence of insect venoms' ability to target a broader range of disease targets like cancer, diabetes, analgesics, autoimmune disease, and antimicrobial effects besides modulation of ion channels at the early stages of drug development^12–14^ The biochemical composition of bee venom (BV), which is produced by female worker bees, is known to contain a variety of active ingredients, that include (i) enzymes like phospholipase A2 (PLA2) and hyaluronidase ; (ii) peptides such as melittin, apamin, mast cell degranulating (MCD) peptide, and adolapin; and (iii) amino acids and volatile substances. Interestingly, bee venom has demonstrated advantageous antiviral capabilities against HIV, comparable to other animal venoms^15^.

Apitoxin is one of the most vital chemicals that bees generate. These insects' abdomens have glands that create complicated chemicals. Apitoxin is 88% water, while the remaining 12% comprises peptides, including apamin and secapin, hyaluronidase, phospholipase A2, histamine, melittin, and others^16,17^.

Another essential component of apitoxin is melittin. It consists of 26 amino acid residues that exhibit amphipathic properties. Melittin interacts with lipid membranes through these residues, making erythrocytes and other cell membranes more permeable. According to Lima and Brochetto-Braga^16^, these amino acids make up around 50% of the apitoxin produced by bees of the species *Apis mellifera* ^18^. The two main components of BV (melittin and PLA2) are recognized individually and together to have antimicrobial properties and can be used as complementary antibacterial medicines besides antiviral effects^15,18^.

Several enveloped and non-enveloped viruses, including vesicular stomatitis (VSV), herpes simplex (HSV), coxsackie (H3), respiratory syncytial (RSV), and influenza A subtype (H1N1, in vivo study), are significantly inhibited by BV and its constituents^19^. Moreover, BV and its component melittin can enhance immunity against porcine reproductive and respiratory syndrome viruses (PRRSV)^19^. Melittin is cytotoxic and may cause cell lysis, as demonstrated by the lysis of human erythrocytes and other peptides^20^. Furthermore, Current research confirms direct interaction between bee venom (BV) and the viral surface. However, the precise mechanisms underlying the antiviral activity of BV and its key component, melittin, remain elusive^21,22^. Additionally, BV and its components can activate type I interferon (IFN), which prevents replication of virus in the host cell^15^.

Wasp venom (WV) peptides and their analogs, such as mastoparan, decoralin, anoplin, polybia-CP, and polydim-I, are now made via solid-phase peptide synthesis. Wasp venom has the potential to be a cutting-edge natural source for the creation of novel medicines and new drug development agents^10^. The WV derivative directly disrupts the lipid envelope structure of five families of enveloped viruses to exhibit broad-spectrum antiviral efficacy against those viruses in vitro. However, to prove its therapeutic value, more research is required^9^.

Mastoparan has 14 amino acid residues and is a membrane-active amphipathic peptide. It is abundant in basic and hydrophobic residues that combine to generate amphipathic helical structures, which can result in membrane holes^9^.

Considering the antimicrobial activities, mastoparan has a wide range of biological impacts, such as injection into the membrane bilayer, which results in membrane destabilization and subsequent lysis, or direct reaction with G proteins on the cytoplasmic face, which disturbs transmembrane signaling^9^.

The venom of the Oriental hornet (*Vespa orientalis*), demonstrated a pronounced inhibitory effect on Hepatitis C virus (HCV) infectivity with a 50% inhibitory concentration (IC_50_) of 10 ng/mL. Notably, this antiviral activity occurred at concentrations significantly lower than the 50% cytotoxic concentration (CC_50_) of 11,000 ng/mL, indicating a wide therapeutic window. However, subsequent analysis revealed that WV exerted no inhibitory effect on the replication of established HCV infections. Therefore, the mechanism of action appears to be limited to the early stages of viral entry. This finding suggests that WV may offer potential as a prophylactic agent against HCV, warranting further investigation^23^. Similarly, the mastoparan-derived peptide MP7-NH2 exhibited potent direct inactivation of various enveloped viruses. This selectivity for enveloped viruses suggests that MP7-NH2 targets a component of the viral envelope, likely the lipid bilayer. Furthermore, pre-treatment of cells with MP7-NH2 failed to reduce the subsequent recovery of infectious virus, further supporting the hypothesis that its primary mechanism of action is direct inactivation of extracellular virions^24^.

The in vitro antiviral properties of insect venom against SARS-CoV-2 have not been reported. Nevertheless, in silico studies focusing on bee venom were conducted, suggesting the need for further in vitro and in vivo investigations^25^.

This work aimed to search for antiviral substitutes targeting the S protein that are low or completely free of diverse effects, which is an urgent need. In this context, Hymenoptera venom, specifically bee and wasp venom, encapsulates diverse exotic constituents, implying an extensive reservoir of potential antiviral agents, as indicated by prior literature documenting their antiviral activity. Moreover, the control of SARS-CoV-2 reproduction and inhibition of its protein synthesis was investigated by assessing the antiviral properties of Hymenoptera venom, explicitly targeting the inhibitory activity on nucleocapsid and spike viral proteins in vitro.

## Results

Evaluation of the antiviral activity of Hymenoptera venom against SARS-CoV-2 using Ortho VITROS® Immunodiagnostic System:

Nucleocapsid protein antigens from SARS-CoV-2 were quantitatively detected in nasopharyngeal swab (NP) specimens using a fully automated immunoassay on the VITROS Immunodiagnostic and VITROS Integrated Systems. The results in Tables 1 and 2 are presented with a numerical signal-to-cutoff (S/CO) value. When comparing the S/CO values of NP samples treated with bee and wasp venoms to untreated NP samples, a noticeable decrease was observed in the S/CO values of the treated samples.

**Table 1:** Signal to cutoff (S/Co) values to sars-covid-2 samples post treated by bee venom of different concentration at interval time.

	Bee venom		
	1 mg	2 mg	2.5 mg
	Mean ± SD	Mean ± SD	Mean ± SD
Control	45.75 ± 20.01	56.63 ± 26.6	48.9 ± 32.12
after 3 h	27.35 ± 10.61	37.37 ± 17.61	25.2 ± 20.36
% of inhibition virus 3 h	39.14 ± 4.61	34.03 ± 0.41	51.21 ± 7.52
after 6 h	22.43 ± 7.05	30.77 ± 17.48	12.48 ± 13.65
% of inhibition virus 6 h	48.84 ± 6.59	48.24 ± 8.22	81.5 ± 15.85
after 24 h	14.58 ± 7.78	12.81 ± 12.38	3.14 ± 2.93
% of inhibition virus 24 h	67.43 ± 9.73	82.25 ± 15.63	94.99 ± 3.6
after 48 h	5.78 ± 2.6	5.44 ± 5.73	1.01 ± 1.47
% of inhibition virus 48 h	84.1 ± 11.25	92.54 ± 7.09	98.65 ± 1.56

Data were represented as mean ± SD. ANOVA test showed a significant difference among means (*P* < 0.001).

**Table 2:** Signal to cutoff (S/Co) values to sars-covid-2 samples post treated by wasp venom of different concentration at interval time.

	Wasp venom		
	1 mg	1.5 mg	2 mg
	Mean ± SD	Mean ± SD	Mean ± SD
Control	88.13 ± 36.77	120 ± 23.07	128.33 ± 8.74
after 3 h	20.48 ± 10.46	52.77 ± 3.96	20 ± 3.61
% of inhibition virus 3 h	75.41 ± 8.72	55.31 ± 5.95	84.3 ± 3.58
after 6 h	13.05 ± 5.85	20.73 ± 6.29	6.36 ± 3.69
% of inhibition virus 6 h	83.82 ± 6.44	82.18 ± 6.68	94.96 ± 3.19
after 24 h	4.06 ± 1.29	0.73 ± 1.01	1.29 ± 1.91
% of inhibition virus 24 h	94.69 ± 2.61	99.31 ± 1.01	98.94 ± 1.59
after 48 h	2.04 ± 0.36	0.4 ± 0.2	0.32 ± 0.03
% of inhibition virus 48 h	97.23 ± 1.54	99.72 ± 0.11	99.75 ± 0.03

Data were represented as mean ± SD. ANOVA test showed a significant difference among means (*P* < 0.001).

**Figure 1:**
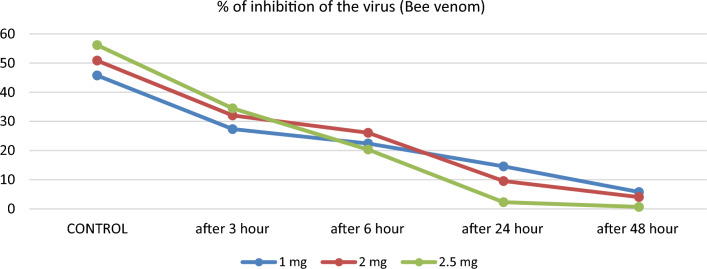
The inhibition in neucleocapsid protein (viral load) of SARS-CoV-2after treating by bee venom of different concentration at different interval time.

After incubation at 37 °C for 3, 6, 24, and 48 h, the NP samples treated with bee venom at a concentration of 1 mg/mL showed a significant decrease (P < 0.001) in SARS-CoV-2 antigen titer compared to the S/CO value before treatment. Similarly, the NP samples treated with bee venom at a 2 mg/mL concentration recorded lower values than the S/CO value before treatment. A high depletion rate of virus antigen titer for the concentration of 2.5 mg/mL of bee venom was recorded. Table 1 and Fig. 1 provide the numerical S/CO values and the percentage of inhibition for each time interval and concentration, demonstrating that the S/CO values of NP samples treated with bee venom were significantly different across all time intervals (*P* < 0.001).

For the NP samples treated with wasp venom at a concentration of 1 mg/mL, a significant decrease (P < 0.001) in SARS-CoV-2 antigen titer was observed after 3, 6, 24, and 48 h of incubation at 37 °C. The depletion rate of the virus antigen titer was higher for the concentration of 1.5 mg/mL of wasp venom, with the highest depletion rate observed at a concentration of 2 mg/mL of wasp venom compared to the S/CO value before treatment. The S/CO values of NP samples treated with wasp venom significantly differed across all time intervals (*P* < 0.001), except for 24 and 48 h, where the detection limit was less than 1. Table 2 displays the S/CO values for SARS-CoV-2 samples treated with wasp venom, along with the percentage of inhibition.

**Figure 2:**
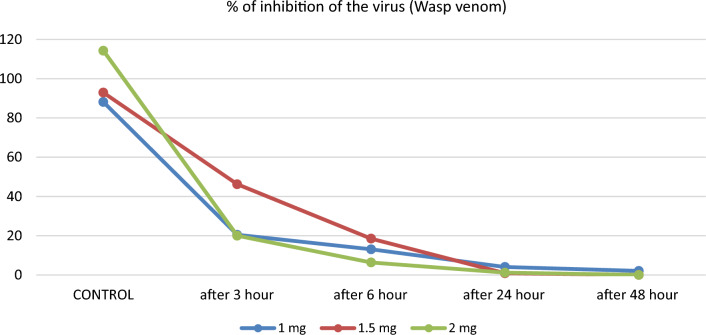
The inhibition in neucleocapsid protein (viral load) of SARS-CoV-2 after treating by wasp venom of different concentration at different interval time.

When comparing the concentration of 1 mg in both bee and wasp venom, it was observed that the percent of inhibition reached 97.23 ± 1.54 for wasp venom (Fig. 2), while it reached 84.1 ± 11.25 for bee venom (Fig. 1). Almost complete inhibition, around 99.72 ± 0.11, was achieved at 1.5 mg of wasp venom after 48 h (Fig. 2). In contrast, bee venom recorded a comparable inhibition percentage of approximately 98.65 ± 1.56, but at a higher concentration of 2.5 mg (Fig. 1).

**Figure 3:**
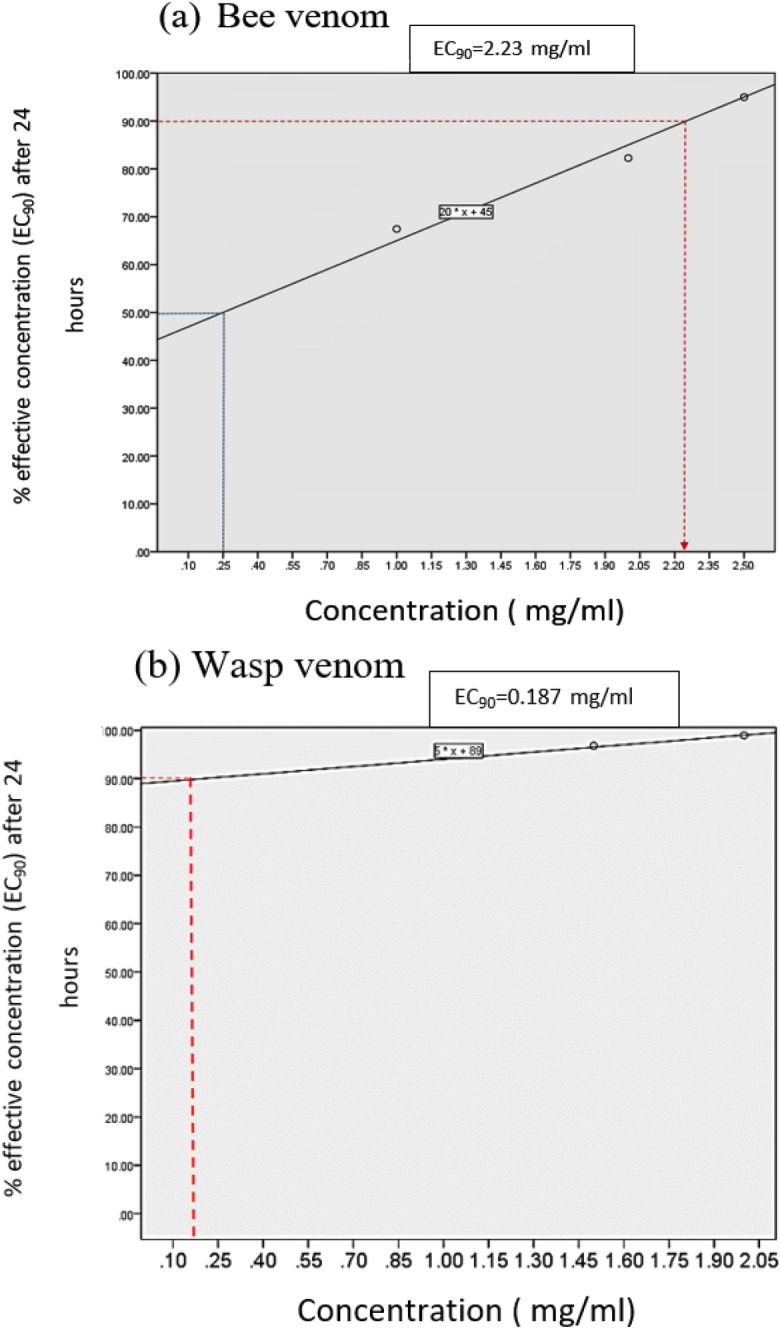
The EC_90_ of a graded S/Co values response curve showed the concentration of the venom where 90% of its maximal antigen depletion is observed after 24 h exposure duration recording 2.23 mg/ml for bee venom (**a**) and 0.187 mg/ml for wasp venom (**b**).

The EC_90_ for bee venom was determined to be 2.23 mg/mL, indicating the concentration at which 90% of its maximal antigen depletion is observed after 24-h exposure time (Fig. 3a). The EC_90_ for wasp venom was 0.184 mg/mL after 24 h (Fig. 3b). It is evident from the data that the effectiveness of wasp venom is higher than that of bee venom, and both venoms demonstrate potent efficiency in reducing antigen titer. Consequently, a cytotoxicity test was conducted on Vero E6 cells to assess the higher efficiency of wasp venom in addition to plaque reduction assay and in silico docking-based screening.

### Evaluation of cytotoxicity of *Vespaorientalis* venom

**Figure 4:**
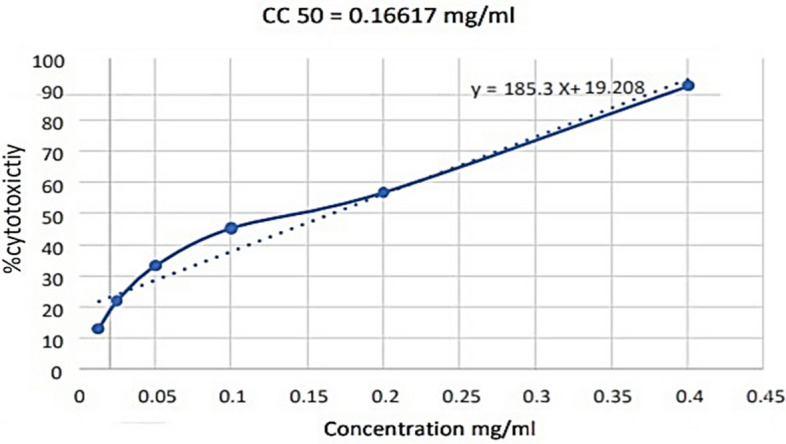
Cytotoxicity percentage of different concentration of the wasp venom against Vero E6 cells showing Half-maximum Cytotoxic concentration (CC_50_).

The cytotoxicity of *Vespa orientalis* venom was evaluated on Vero E6 cells 48 h post-treatment using MTT assay. This finding demonstrates that the *Vespa orientalis* venom can affect the viability percentage of cells in a concentration-dependent manner, as shown in Fig. 4. The concentrations that inhibit 50% of the cell growth were 0.16617 mg/mL for Vero E6 cells.

The MTT assay revealed the percentage of cytotoxicity, measured as the absorbance of cells with treatment compared to untreated cells, indicating a cytotoxic concentration (CC_50_) of 0.16617 mg/mL.

### 3- Plaque reduction assay (SARS-CoV-2)

Table 3 presents the reduction in viral count compared to the initial viral count and the inhibition percentages for the tested wasp venom. The IC_50_ of the wasp venom was determined to be 0.208 mg/mL, with the viral count at 50% inhibition recorded as 2.5 × 10^4 ^PFU/mL.

**Table 3:** Anti-SARS-CoV2 activity of the wasp venom (Plaque Reduction Assay).

Conc (mg/ml)	Initial viral count (PFU/ml)	Viral count (PFU/ml)	Inhibition %	IC _50_
0.2	5 × 10^4^	2.6 × 10^4^	48	0.208
0.1		3.15 × 10^4^	37	
0.05		3.9 × 10^4^	22	
0.025		4.05 × 10^4^	19	

IC_50_: 50% inhibitory concentration.

### 4-Molecular modeling of interactions of wasp venom (Mastoparan) with spike -ACE 2 of SARS-CoV-2

Molecular modeling was conducted to explore the interactions of wasp venom, specifically mastoparan, with the spike-ACE2 complex of SARS-CoV-2. Computational software was employed for molecular docking studies to assess the binding affinities of mastoparan towards the proposed target. The modeling process involved the preparation of the spike angiotensin-converting enzyme 2 (spike-ACE2), the receptor and the ligand (Mastoparan), and molecular docking principles were applied. The best-docked pose, determined by the lowest glide score value, was recorded to measure the potential interaction strength.

**Figure 5:**
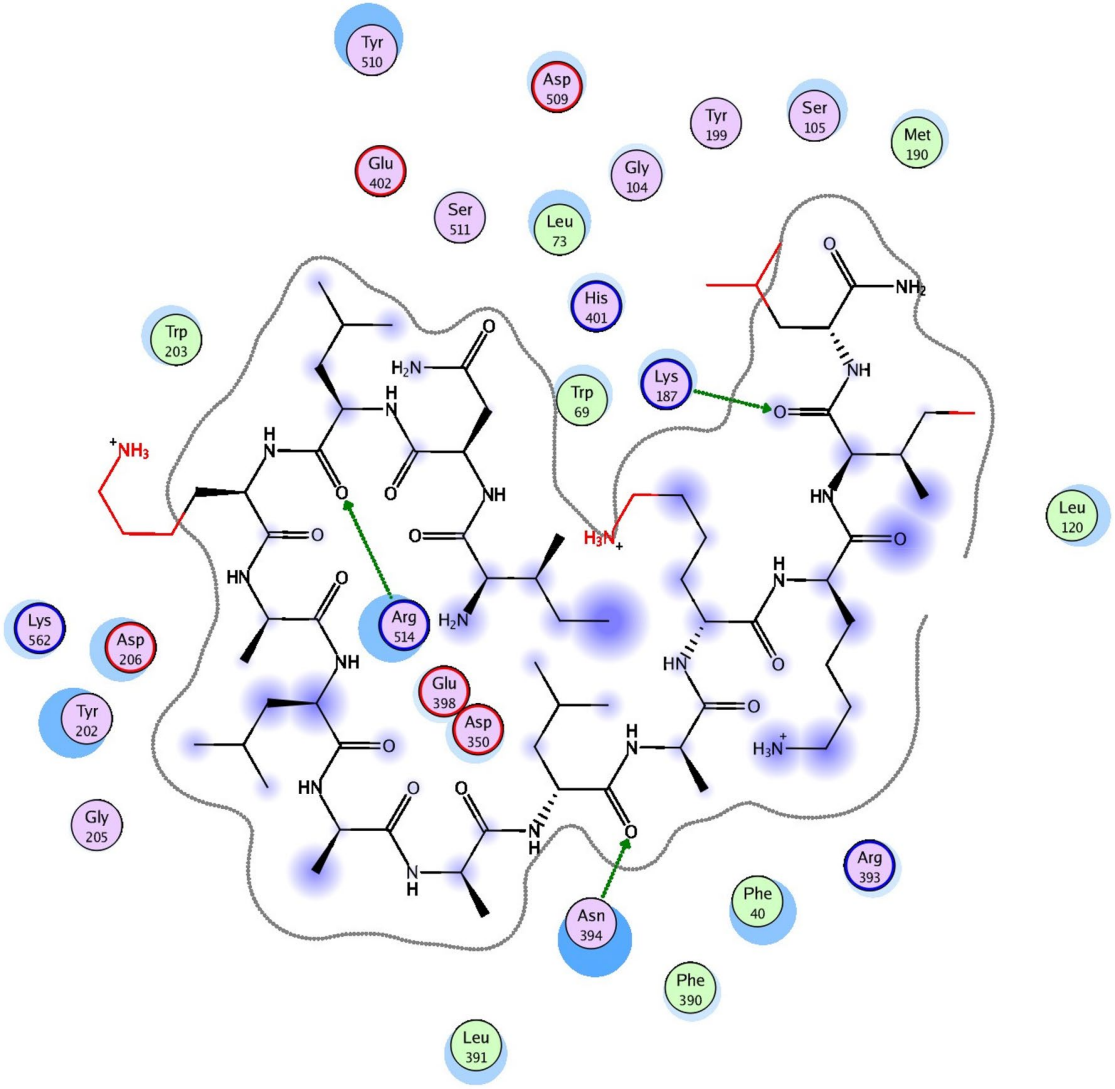
The 2D structure of the ACE2 protein with the wasp venom (Mastoparan) in the pocket of the protein.

The molecular docking results revealed interactions of wasp venom (Mastoparan) with spike-ACE2 of SARS-CoV-2, where three poses exhibited better score values with the ability to form interactions with GLU 398, LYS 187, ASN 394, and ARG 514 in the binding pocket (Table 4). The two-dimensional interaction diagram between mastoparan and ACE2 residues illustrated the presence of four crucial hydrogen bonds (Fig. 5). The overall binding affinity was scored at -7.5.

**Table 4:** Molecular docking results of wasp venom (Mastoparan) with Spike -ACE 2 of SARS-CoV-2.

Ligand	Receptor	Residue	TYPE	Distance	Binding energy (Kcal/mol)
H 5897	OE 3091	GLU 398	H-don	1.88	− 7.5
O 6040	NZ 1379	LYS 187	H-acc	2.61	
O 5967	ND 3065	ASN 394	H-acc	2.29	
O 5877	NH 4046	ARG 514	H-acc	2.42

## Discussion

The limitations of current COVID-19 therapeutics necessitate the exploration of alternative and innovative therapeutic strategies.^26,27^ It is essential to identify novel host genes or proteins that contribute to COVID-19 pathogenesis.

Exploring animal venoms can provide valuable insights for developing new therapeutic treatments.^12^. Insect venoms represent a blend of bioactive components with diverse physiological actions, rendering them promising candidates for target-specific drug discovery^12,28^^.^

Despite the completion of numerous research works on insect venom and its therapeutic potential, only a few studies have been published on the practical application of insect venom as a bioavailable drug.

Concerning COVID-19, a single published study has conducted in silico analysis of bee venom^25^; another preprint data , which remains unpublished, has demonstrated in silico analysis of wasp venom^29^. Both studies recommend further in vitro experiments^25,29^.

However, no in vitro study has evaluated the effects of both bee and wasp venom against SARS-CoV. Additionally, both in silico analyses and cytotoxicity assays are lacking. This study aimed to fill this gap by evaluating the efficacy of both bee and wasp venom against SARS-CoV-2 in vitro and identifying the most effective one followed by in silico analysis and cytotoxicity assessment for the most potent venom.

The Ortho VITROS® Immunodiagnostic System, originally designed for clinical diagnostic purposes, has demonstrated a high correlation in measuring the antiviral activity of Hymenoptera venom in vitro. This is achieved by detecting and quantifying SARS-CoV-2 nucleocapsid antigen titers in venom-treated samples with varying concentrations and exposure times, relying on the applied S/CO values. All samples testing positive with real-time RT-PCR (Ct values below 30.0) were consistently positive in nucleocapsid antigen detection. The observed continuous decline in S/CO values with increasing venom concentration and exposure time is evidence of the anti-SARS-CoV-2 activity of Hymenoptera venom. Results indicated that wasp venom exhibited a higher antiviral effect compared to bee venom; at 48 h, the S/CO value decreased from 128.33 ± 8.74 of control non-treated samples to 0.32 ± 0.03 for 2 mg of wasp venom-treated samples, with a percent inhibition of 99.75 ± 0.03 (Table 2). In contrast, the same concentration of 2 mg bee venom, despite a lower viral load (56.63 ± 26.6) than wasp venom, resulted in a S/CO value of 5.44 ± 5.73 with a percent inhibition of 92.54 ± 7.09 (Table 1). Additionally, the 90% effective concentrations (EC_90_) of wasp venom causing a 90% antigen depletion effect was recorded as 0.184 mg/mL, which is less than 12 times the concentration required by bee venom (2.23 mg/mL) to achieve a similar level of 90% depletion of SARS-CoV-2 nucleocapsid antigen.

The estimated IC_50_ values of 223 μg/mL and 184 μg/mL for bee and wasp venom, respectively, are quite high compared to the reported 10 ng/ml IC_50_ for Oriental hornet venom against Hepatitis C virus^24^ . However, the antiviral potency can vary significantly depending on the specific virus and target cell types used in the assays. The high IC_50_ values observed for bee and wasp venoms may be specific to the viruses and cells tested in this study especially, the lower concentrations below the tested ones didn’t give any inhibition even after prolonged incubation of 72 h and no previous guide studies of antiviral activity of any crude venom or venom bioactive molecules against SARS-CoV-2 is available except **Al-**Rabia, et al., 2021^30^ which use of Sitagliptin (SIT) with melittin (MEL) nano-conjugate against SARS-CoV-2 virus **(**note that, Sitagliptin (SIT) belongs to the drug class dipeptidyl peptidase-4 (DPP-4) inhibitor, medication for Type 2 diabetes. Also, Melittin is the major peptide representing approximately 50% of the dry weight of bee venom)^30^, SIT-MEL nano-conjugates showed a potent antiviral effect against the SARS-CoV-2 virus with IC_50_ = 8.43 µM, the value of 8.43 µM equal to 0.01686 mg/ml meaning that SIT-MEL nano-conjugates more potent 10 times than the crude wasp venom (more potent crude venom), this difference is normal and isn’t high difference as Mellittin is the primary antiviral compound, and by isolating and concentrating it, the inhibitory potency is increased compared to the whole crude venom mixture especially when conjugated with another antiviral compound like Sitagliptin moreover, the nanoformulation of both melittin and Sitagliptin can enhance its delivery, stability, and bioavailability compared to the free forms so, the importance of separating and characterizing bioactive compounds from crude venom in the future is highlighted, as well as the potential of using nanotechnology to improve the antiviral efficacy, especially against SARS-CoV-2.

It is likely that Hymenoptera venom contains compounds that induce destruction to the SARS-CoV-2 nucleocapsid antigen, resulting in low S/CO values. Notably, wasp venom demonstrated 12 times more potent virucidal activity against SARS-CoV-2 than bee venom. This variation in antiviral activity could be attributed to the differences in venom composition between the two insect species. Mastoparan, a unique and exclusive peptide found in wasp venom, has been shown to cause pores and disrupt viral lipid envelopes^31^. Consequently, it is plausible that the observed virucidal activity against SARS-CoV-2 in wasp venom may be attributed to mastoparan. Preceding studies indicate that mastoparan analogues, specifically mastoparan-7, display broad-spectrum antiviral activity against enveloped viruses representing six distinct families as observed in in vitro assays ^31,32^.

The phospholipases, another component found in Vespoid wasp crude venom, can disrupt the packing of phospholipids in various biological membranes. This disruption can lead to the formation of pores and the lysis of cells by catalyzing the hydrolysis of ester bonds in specific positions of certain phospholipids^33,34^^.^ Vespoid phospholipase A2 (PLA2) demonstrates potent cytolytic actions, indicating its potential role in the antiviral activity against HCV^23^. Mastoparans, found in the wasp venom, can stimulate PLA2 from different sources^35^. PLA2 and mastoparan may work together to induce virucidal activity by forming a complex in the wasp venom. In silico investigations have shown that mastoparan interacts with various sites on ACE2, which play roles in direct or indirect contact with the ACE2 receptor. This interaction suggests a potential blocking effect on the functional ACE2 receptor complex.

The investigated compounds, particularly wasp venom, demonstrate potential efficacy as a potent inhibitor of ACE, suggesting its possible use as a novel treatment for inhibiting COVID-19 cell entry and preventing associated inflammatory complications. MTT results indicated low toxicity of wasp venom to normal cells, making it a potential candidate for antiviral applications pending further in vivo assays. Notably, the effective dose that inhibited 50% of the virus was determined to be 0.16617 mg/mL.

The antiviral effect of crude wasp venom may be attributed to one or more common or non-common molecules or toxin complexes present in the venom, particularly mastoparan. Further studies are required to identify and characterize the active compound(s) responsible for the antiviral activity of wasp venom.

### In conclusion

Further research is warranted to use standard venomics techniques to isolate, identify, and fully characterize any promising SARS-CoV-2 inhibitors discovered in venom samples. Additional in vitro experiments using lung cell lines also need to be conducted to explore how these venoms might impact viral processes. All of this will help validate the potential of wasp and bee venom to treat SARS-CoV-2 in vivo, providing a basis for developing new antiviral drugs. Given wasp venom's multiple targets and promising inhibitory effects against SARS-CoV-2 based on in silico studies, it has potential to contribute to antiviral therapies. Considering the dynamic nature of COVID-19 treatments, future research should aim to compare the findings of this study with the prevailing treatment methods at that time.

## Methods

### Venoms

The bee *Apis mellifera* and the wasp *Vespa orientalis* venoms were extracted at the apiculture , Plant Protection Research Institute, Dokki, Giza, Egypt, by electrical chock method according to (Mohanny,et al. 2013)^36^. Both venoms were powder and solubilized in nuclease-free water. Bee venom was divided into 3 groups of 1, 2, and 2.5 mg/mL concentrations. Wasp venom was divided into 3 groups of 1, 1.5, and 2 mg/mL concentrations.

### Viral stock

The specimens collected comprised dry swap nasopharyngeal samples, obtained following CDC guidelines and adhering to proper infection control and personal protective equipment (PPE) protocols^1–3^. The samples were collected from 20 Egyptian patients diagnosed with severe acute respiratory syndrome, all confirmed positive for SARS-CoV-2 through real-time PCR with Ct values below 30.0. The study was conducted at BSL-3 Lab—Armed Force Labs for Medical Research, Egypt. The dry swaps were stored in Vacuette virus stabilization tubes, which are made of PET with a pre-defined volume of transport media (phosphate buffered saline solution, 3 mL) at a pH of 7.4, allowing for the storage of the SARS-CoV-2 swab specimens viable for up to 72 h at 4 °C^37^.

Sample preparation.

The 20 positive SARS-CoV-2 nasopharyngeal samples (NS) were evenly divided, with 10 treated using bee venom (BV) and the remaining 10 treated with wasp venom (WV). Each nasopharyngeal sample (transport media 3 mL contain infected swap) was divided equally into control and test groups. Approximately 200 µL from the control sample was combined with 200 µL of clean transport media and designated as control samples, deemed viable for up to 72 h at 4 °C. New control nasopharyngeal samples were collected and measured alongside the test samples at 3, 6, 48, and 72 h intervals. For each test sample, 200 µL was combined with 200 µL of venom at varying concentrations for BV and WV in separate Wasserman tubes. The sample mixtures (400 µL each) were then incubated at 37 °C at 3, 6, 48, and 72-h intervals. In the case of BV treatment, out of the ten nasopharyngeal samples, three were treated with 1 mg/mL, three with 2 mg/mL, and the remaining four with 2.5 mg/mL. Similarly, three of the ten WV-treated nasopharyngeal samples were treated with 1 mg/mL, three with 1.5 mg/mL, and the remaining four with 2 mg/mL. Thus, each venom type has three groups with different concentrations.

Serological identification of SARS-CoV-2 antiviral activity of Hymenoptera venom using Ortho VITROS® Immunodiagnostic System.

The Ortho VITROS® SARS-CoV-2 antigen test is commonly employed to detect SARS-CoV-2 infection levels precisely. This is achieved by extracting the viral load from positive swab samples of SARS-CoV-2 placed in suitable transport media. It targets the nucleocapsid protein, the superabundant protein of the SARS-CoV-2 virus. Both the control and incubated venom-treated sample mixtures (400 µL each) were combined with 100 µL of the VITROS SARS-CoV-2 antigen extraction buffer, which contains ≥ 0.0015 – < 0.06% of a mixture of 3(2H)-isothiazolone, 5-chloro-2-methyl- with 2-methyl-3(2H)-isothiazolone, in an Ortho VITROS® cuvette. Subsequently, the Ortho VITROS®3600 Immunodiagnostic System was operated 45 min before readings were taken. The SARS-CoV-2 nucleocapsid antigen immunoassay is a two-stage chemiluminesce reaction. In the first stage, SARS-CoV-2 nucleocapsid antigens in the sample bind with monoclonal anti-SARS-CoV-2 antibodies that are coated on the wells of a microplate. The unbound sample is then washed away. In the next stage, horseradish peroxidase (HRP)-labeled monoclonal anti-SARS-CoV-2 antibodies are added. These antibodies bind to any SARS-CoV-2 nucleocapsid antigens that were captured in the first stage. Unbound conjugate is removed by washing. The amount of bound HRP conjugate is measured by a luminescent reaction. A reagent including luminogenic substrates and an electron transfer agent is added to the wells. The HRP in the bound conjugate catalyzes the oxidation of the luminol derivative, producing light. The electron transfer agent increases the light produced and prolongs its emission. The signal-to-cutoff ratio is proportional to the amount of SARS-CoV-2 antigen in the sample^38^. The evaluation was based on the 90% effective concentration (EC_90_) to identify the more effective venom. EC_90_ is the concentration necessary to achieve a 90% effect, specifically antigen depletion in this context. It is a commonly used measure of substance or drug potency, with EC_90_ values considered a more indicative marker than EC_50_ for the concentrations required to decrease SARS-CoV-2 antigen levels^32^.

EC_90_ values were determined using signal-to-cutoff (S/Co) data after 24 h, which was determined using logarithmic interpolation. All data obtained were analyzed, and all graphical representations were performed using IBM SPSS version 25.0.

### Cytotoxicity assay of wasp venom

The cytotoxic activity of wasp venom, known for its potency, was evaluated on Vero E6 cells utilizing the 3-(4,5-dimethylthiazol-2-yl)-2,5-diphenyltetrazolium bromide (MTT) assay with slight modifications^39^ . Wasp venom was diluted in Dulbecco's Modified Eagle Medium (DMEM), while stock solutions of wasp venom were prepared in 10% DMSO in double-distilled water. Vero E6 cells were seeded in 96-well plates at a density of 3 × 103 cells/mL (100 µL/well) for incubation 24 h at 37 °C under 5% CO_2_. Subsequently, cells were exposed to varying concentrations of the wasp venom in triplicate for an additional 24 h. After treatment, the supernatant was eliminated, and cell monolayers were meticulously washed with sterile phosphate-buffered saline (PBS) on three times. Then, 20 µL of a 5 mg/mL MTT stock solution was added to each well, followed by 24 h incubation at 37 °C and subsequent aspiration of the medium. Finally, the resulting formazan crystals in each well were dissolved using 200 µL of acidified isopropanol (0.04 M HCl in absolute isopropanol) and quantified.

This meticulously designed experiment enabled the assessment of wasp venom's cytotoxic activity on Vero E6 cells, facilitating comparisons with the effects of other test compounds under controlled conditions.

The absorbance of formazan solutions was measured at λmax = 540 nm with 620 nm as a reference wavelength using a multi-well plate reader. The percentage of cytotoxicity compared to the untreated cells was determined with the following Eq.^40^.

%C*ytotoxicity* = (*absorbance of cells wit*h*out treatment* − *absorbance of cells wit*h *treatment*)100/ *absorbance of cells wit*h*out treatmen.*

The plot of % cytotoxicity versus sample concentration was used to calculate the concentration which exhibited 50% cytotoxicity (CC_50_).

Plaque reduction assay (SARS-CoV2).

This study employed a plaque reduction assay in Vero E6 cells to evaluate the anti-SARS-CoV-2 potential of various test compounds. The methodology adhered to the protocol established by Hayden et al. (2020)^41^.

Vero E6 cells were cultivated in six-well plates for 24 h at 37 °C to achieve optimal confluence. SARS-CoV2 stocks were diluted to yield approximately 10^3^ plaque-forming units (PFU)/well, ensuring consistent viral challenge. Diluted virus was pre-incubated with varying concentrations of wasp venom (within their safe use range) for a 1 h at 37 °C, facilitating potential virus-compound interaction. The pre-incubated virus-compound mixture was inoculated onto the Vero E6 cell monolayer, followed by a 1-h incubation at 37 °C to permit viral attachment. An overlay containing DMEM supplemented with 2% agarose and the test compounds was added to each well, solidifying the mixture and preventing further viral spread. Plates were incubated at 37 °C for 3–4 days, allowing efficient viral replication and the formation of distinct cytopathic plaques indicative of productive infection. Following incubation, 10% formalin was applied for 2 h to fix the infected cells, preserving their morphology for analysis. Subsequently, 0.1% crystal violet in distilled water was used to stain the fixed cells, highlighting the formed plaques for visualization and enumeration. Untreated virus-inoculated wells served as a control , establishing the baseline level of viral plaque formation in the absence of wasp venom. Visible plaques in each well were meticulously counted, and the percentage reduction in plaque formation compared to control wells was calculated using the standard formula:

*% Inhibition* = *{(viral count “untreated”—viral count “treated”)/(viral count “untreated”)}* × *100*

Analysis: By quantifying the reduction in plaque formation compared to untreated controls, this experiment provided a quantitative assessment of the antiviral efficacy of the wasp venom against SARS-CoV2 within the Vero E6 cell model.

### In silico study

The in silico study was performed to predict the synergistic effect of the most potent venom (wasp venom) against spike-ACE 2 of SARS-CoV-2.

### Molecular docking of ACE 2 protein and ligands

The spike -ACE 2 – receptor protein sequence was downloaded from Protein Data Bank (https://www.rcsb.org) under accession numbers 5FUC. For the pre-docking process, all H2O molecules from the PDB compound of the proteins and ligands were eliminated while hydrogen atoms were added to the target proteins. The docking system was constructed using AutoDock Vina.

The 3D structure of mastoparan is selected and severally downloaded from PubChem (https://pubchem.ncbi.nlm.nih.gov/) in SDF format, then changed to MOL2 format by using OpenBabel (http://openbabel.org/wiki/Main_Page), (PubChem 6,324,633).

### Virtual screening and docking protocol

The study focused on investigating the spike-ACE2 interaction, examining conservative residues for the binding of the spike protein (accession 6MOJ) with ACE as a control. Additionally, the interaction mechanism was explored by assessing ACE2 with mastoparan ligands, which exhibited good binding affinity.

The docking and scoring of each compound from the previous dataset were used to perform virtual screening. Research was conducted to predict the affinities and modalities of binding each chemical component using docking to spike -ACE 2 (experimental proteins). AutoDock Vina can acquire the docking parameter from docking programs, allowing docking for a collection of ligands to be performed on a single protein. Considering these factors, many structure and protein targets were tested. The docked poses of high-scoring compounds with low RMSD had to be seen to assess their structural integrity because many ligands were docked in different confirmations. Therefore, the lowest poses for the compounds had to be eliminated first using filtration for ligand verification. Subsequently, the ligand exploration was conducted utilizing tools such as Molecular Operating Environment(https://www.chemcomp.com/Products.htm),SAMSON (https://www.samson-connect.net), and Discovery Studio Visualizer (https://discover.3ds.com/discovery-studio-visualizer-download). These platforms were employed to investigate the ligand qualities essential for reaching the functional protein domain in humans.

All methods were carried out in accordance with relevant guidelines and regulations. The ethical approval was obtained from the research ethics committee, Faculty of Science, Ain Shams University.

### Statistical analysis

Data were analyzed with one-way ANOVA with a Tukey's test for multiple comparisons. *P* < 0.05 and < 0.005 were considered statistically significant. All analyses were performed with IBM SPSS version 25.0.

### Ethical approval

This research paper was approved by the research ethics committee from the Faculty of Science, Ain Shams University which approved the experiments, including any relevant details under the code (ASU-SCI/ENTO/2022/10/17).

### Informed consent

The informed consent was obtained from all subjects and / or their legal guardian(s).

## Data Availability

All data generated or analyzed during this study are included in this published article.

## References

[1] Rothan, H. A. & Byrareddy, S. N. Since January 2020 Elsevier has created a COVID-19 resource centre with free information in English and Mandarin on the novel coronavirus COVID- 19. The COVID-19 resource centre is hosted on Elsevier Connect, the company ’ s public news and information. (2020).

[2] Burbelo PD (2020). Sensitivity in detection of antibodies to nucleocapsid and spike proteins of severe acute respiratory syndrome coronavirus 2 in patients with coronavirus disease 2019. J. Infect. Dis..

[3] Tombuloglu H (2022). Multiplex real-time RT-PCR method for the diagnosis of SARS-CoV-2 by targeting viral N, RdRP and human RP genes. Sci. Rep..

[4] Razai MS (2021). COVID-19 vaccine hesitancy: The five Cs to tackle behavioural and sociodemographic factors. J. R. Soc. Med..

[5] Essa EE (2022). The antibacterial activity of Egyptian Wasp chitosan-based nanoparticles against important antibiotic-resistant pathogens. Molecules.

[6] Mahmoud F (2022). Double-coated microencapsulation of honeybee endogenous probiotics as a new strategy for the biocontrol of the American foulbrood disease. Egypt. J. Biol. Pest Control.

[7] Zulfiqar B, Shelper TB, Avery VM (2017). Leishmaniasis drug discovery: Recent progress and challenges in assay development. Drug Discov. Today.

[8] Alves RRN, Albuquerque UP, Alves RRN, Albuquerque UP (2013). Animals as a Source of Drugs: Bioprospecting and Biodiversity Conservation. Animals in Traditional Folk Medicine.

[9] Moreno M, Giralt E (2015). Three valuable peptides from bee and wasp venoms for therapeutic and biotechnological use: Melittin, apamin and mastoparan. Toxins (Basel)..

[10] El-Wahed AA (2021). Wasp venom biochemical components and their potential in biological applications and nanotechnological interventions. Toxins (Basel)..

[11] Santos LD, Pieroni M, Menegasso ARS, Pinto JRAS, Palma MS (2011). A new scenario of bioprospecting of Hymenoptera venoms through proteomic approach. J. Venom. Anim. Toxins Incl. Trop. Dis..

[12] Moran D, Dutta U, Kunnumakkara AB, Daimari E, Deka B (2022). Insect venoms and their bioactive components: A novel therapeutic approach in chronic diseases and cancer. J. Cancer Sci. Clin. Ther..

[13] Minutti-Zanella C, Gil-Leyva EJ, Vergara I (2021). Immunomodulatory properties of molecules from animal venoms. Toxicon.

[14] Magdy H (2023). Isolation of multidrug-resistant helicobacter pylori from wild houseflies musca domestica with a new perspective for the treatment. Vector-Borne Zoonotic Dis..

[15] Wehbe R (2019). Bee venom: Overview of main compounds and bioactivities for therapeutic interests. Molecules..

[16] De Lima PR, Brochetto-Braga MR (2003). Hymenoptera venom review focusing on Apis mellifera. J. Venom. Anim. Toxins Incl. Trop. Dis..

[17] Alia O, Laila M, Al-Daoude A (2013). Antimicrobial effect of melittin isolated from Syrian honeybee (Apismellifera) venom and its wound healing potential. Int. J. Pharm. Sci. Rev. Res..

[18] Leandro LF (2015). Antimicrobial activity of apitoxin, melittin and phospholipase A2 of honey bee (Apis mellifera) venom against oral pathogens. An. Acad. Bras. Cienc..

[19] El-Seedi H (2020). Antimicrobial properties of apis mellifera’s bee venom. Toxins.

[20] Pandey BK (2010). Cell-selective lysis by novel analogues of melittin against human red blood cells and *Escherichia coli*. Biochemistry.

[21] Zhu WL, Nan YH, Hahm K-S, Shin S-Y (2007). Cell selectivity of an antimicrobial peptide melittin diastereomer with D-amino acid in the leucine zipper sequence. BMB Rep..

[22] Carvalho LAC, Machini MT (2013). Hemocidinas derivadas da hemoglobina: Estruturas, propriedades e perspectivas. Quim. Nova.

[23] Sarhan M, El-Bitar AMH, Mohammadein A, Elshehaby M, Hotta H (2021). Virucidal activity of oriental hornet Vespa orientalis venom against hepatitis virus. J. Venom. Anim. Toxins Incl. Trop. Dis..

[24] Sample CJ (2013). A mastoparan-derived peptide has broad-spectrum antiviral activity against enveloped viruses. Peptides.

[25] Abdelfattah EA (2021). Biotechnology approach to perform a computational model of bee venom against COVID-19. J. Egypt. Soc. Parasitol..

[26] Singh DD, Han I, Choi E-H, Yadav DK (2023). A clinical update on SARS-CoV-2: Pathology and development of potential inhibitors. Curr. Issues Mol. Biol..

[27] Robinson PC (2022). COVID-19 therapeutics: Challenges and directions for the future. Proc. Natl. Acad. Sci..

[28] Dossey AT (2010). Insects and their chemical weaponry: New potential for drug discovery. Nat. Prod. Rep..

[29] Kadhim Nimr H (2023). In silico study: Wasp venom peptide as covid 19 antiviral drug. Researchgate. Net.

[30] Al-Rabia M (2021). Repurposing of sitagliptin- melittin optimized nanoformula against SARS-CoV-2; antiviral screening and molecular docking studies. Pharmaceutics.

[31] Higashijima T (1983). Conformational change of mastoparan from wasp venom on binding with phospholipid membrane. FEBS Lett..

[32] Arshad U (2020). Prioritization of anti-SARS-Cov-2 drug repurposing opportunities based on plasma and target site concentrations derived from their established human pharmacokinetics. Clin. Pharmacol. Ther..

[33] Santos LD (2007). Purification, sequencing and structural characterization of the phospholipase A1 from the venom of the social wasp Polybia paulista (Hymenoptera, Vespidae). Toxicon.

[34] Costa H, Palma MS (2000). Agelotoxin: a phospholipase A2 from the venom of the neotropical social wasp cassununga (Agelaia pallipes pallipes) (Hymenoptera-Vespidae). Toxicon.

[35] Argiolas A, Pisano JJ (1983). Facilitation of phospholipase A2 activity by mastoparans, a new class of mast cell degranulating peptides from wasp venom. J. Biol. Chem..

[36] Mohanny, K. M. The Efficacy of a New Modified Apparatus for Collecting Bee Venom in Relation to Some Biological Aspects of Honeybee Colonies. (2013).

[37] In, F. & Diagnostic, V. VACUETTE ® Virus Stabilization Tube. 1–2 (2021).

[38] Vitros SARS-CoV-2 Antigen Reagents. *Biomed. Saf. Stand.* **51**, 77–77 (2021).

[39] Mosmann T (1983). Rapid colorimetric assay for cellular growth and survival: Application to proliferation and cytotoxicity assays. J. Immunol. Methods.

[40] Eiaka, ; 2 El-Bagoury, A. M., El-Nahas & Khodeir, M. H. *Evaluation of the antiviral effect of bee venom on rabies virus*. http://www.bvmj.bu.edu.eg.

[41] Hayden FG, Cote KM, Douglas RG (1980). Plaque inhibition assay for drug susceptibility testing of influenza viruses. Antimicrob. Agents Chemother..

